# Application of smart spectrophotometric methods and artificial neural network for the simultaneous quantitation of olmesartan medoxamil, amlodipine besylate and hydrochlorothiazide in their combined pharmaceutical dosage form

**DOI:** 10.1186/1752-153X-7-22

**Published:** 2013-02-01

**Authors:** Hany W Darwish

**Affiliations:** 1 Department of Pharmaceutical Chemistry, College of Pharmacy, King Saud University, P.O. Box 2457, Riyadh 11451, Saudi Arabia; 2 Department of Analytical Chemistry, Faculty of Pharmacy, Cairo University, Kasr El-Aini Street, ET 11562 Cairo, Egypt

**Keywords:** Olmesartan medoxamil, Amlodipine besylate, Hydrochlorothiazide, Spectrophotometry, Artificial neural network

## Abstract

**Background:**

New, simple and specific spectrophotometric methods and artificial neural network (ANN) were developed and validated in accordance with ICH guidelines for the simultaneous estimation of Olmesartan (OLM), Amlodipine (AML), and Hydrochlorothiazide (HCT) in commercial tablets.

**Results:**

For spectrophotometric methods: First, Amlodipine (AML) was determined by direct spectrophotometry at 359 nm and by application of the ratio subtraction, the AML spectrum was removed from the mixture spectra. Then Hydrochlorothiazide (HCT) was determined directly at 315 nm without interference from Olmesartan medoxamil (OLM) which could be determined using the isoabsorptive method. The calibration curve is linear over the concentration range of 5–40, 2.5-40 and 2–40 μg mL^-1^ for AML, OLM and HCT, respectively. ANN (as a multivariate calibration method) was also applied for the simultaneous determination of the three analytes in their combined pharmaceutical dosage form using spectral region from 230–340 nm.

**Conclusions:**

The proposed methods were successfully applied for the assay of the three analytes in laboratory prepared mixtures and combined pharmaceutical tablets with excellent recoveries. No interference was observed from common pharmaceutical additives. The results were favorably compared with those obtained by a reference spectrophotometric method. The methods are validated according to the ICH guidelines and accuracy, precision and repeatability are found to be within the acceptable limit.

## Background

**Figure 1:**
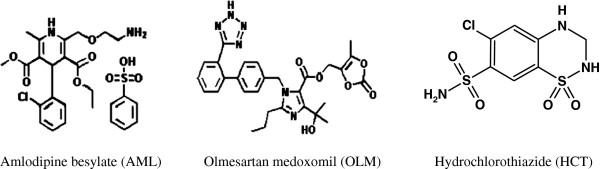
Chemical structures of amlodipine besylate (AML), olmesartan medoxomil (OLM) and hydrochlorothiazide (HCT).

Olmesratan medoxamil (OLM, Figure 1) is chemically known as N-[p-(o-1H-Tetrazol-5-ylphenyl) benzyl]-N-valeryl-L-valine [1]. It is a potent and selective angiotensin AT1 receptor blocker [2], It has been approved for the treatment of hypertension in the United States, Japan and European countries. The drug contains a medoxomil ester moiety which is cleaved rapidly by an endogenous esterase to release the active olmesartan [3]. Amlodipine besylate (AML, Figure 1) is chemically known as 2-[(2-aminoethoxy) methyl]-4-(2-chlorophenyl)-1,4-dihydro-6-methyl-3,5-pyridine carboxylic acid 3-ethyl 5-methyl ester) [1]. It is a dihydropyridine calcium channel blocker used in the treatment of hypertension and angina pectoris [4]. Hydrochlorothiazide (HCT, Figure 1) is chemically known as 6-chloro-3,4-dihydro-2*H*-1,2,4-benzothiadiazine-7-sulphonamide-1,1-dioxide [1]. It is a diuretic of the class of benzothiadiazines widely used in antihypertensive pharmaceutical formulations, alone or in combination with other drugs, which inhibits NaCl transport in distal convoluted tubule and decreases blood pressure [5]. Recently, OLM has been marketed in combination with AML and HCT in tablet dosage form (TRIBENZOR® tablets). The triple combination of OLM, AML and HCT is intended for oral administration for the treatment of hypertension.

Literature survey revealed that AML is official in British Pharmacopoeia [6] and HCT is official in British Pharmacopoeia [6] and United States Pharmacopoeia [7]. Few methods are available for the simultaneous analysis of OLM, AML and HCT combination. These methods include HPLC [8-10] and spectrophotometry [11-13]. Therefore, the aim of this work was directed to the development of simple, sensitive, selective and validated spectrophotometric methods in addition to ANN for the simultaneous determination of OLM, AML and HCT in their combined dosage form. The linearity of response, accuracy, intermediate precision and robustness of the described method for assay of AML, OLM and HCT has been checked.

## Experimental

### Apparatus

A double-beam uv–visible spectrophotometer (shimadzu, japan) model uv-1650 pc with quartz cell of 1 cm path length, connected to an IBM-compatible computer. The spectral bandwidth was 2 nm and wavelength-scanning speed 2800 nm/min. A uv lamp with a short wavelength (254 nm).

### Software

ANN was implemented in Matlab® 7.1.0.246 (R14) using Neural Network toolbox The *t* test, *F* test were performed using Microsoft® Excel. All calculations were performed using intel® core™ i5-2400, 3.10 GHz, 4.00 GB of RAM under Microsoft Windows 7.

### Materials

OLM was obtained from AK Scientific Inc. (CA, USA). AML was obtained from Pfizer Inc. (New York, USA). HCT was kindly supplied by Al-Hekma pharmaceutical Company (Cairo, Egypt). The purities of OLM, AML and HCT were 99.5%, 99.5% and 99.78% respectively. Tribenzor® tablets 40/10/25 (Daiichi Sankyo inc., U.S.A) are label to contain 40 mg of OLM, 10 mg of AML base (equivalent to 13.9 mg of AML) and 25 mg of HCT (Batch number 134809). They were procured from U.S.A. market. Acetonitrile used throughout this study was of analytical grade.

### Preparation of OLM, AML and HCT standard solutions

Stock solutions of OLM (250 μg mL^−1^), AML (200 μg mL^−1^) and HCT (250 μg mL^−1^) were prepared by dissolving 12.5 mg of OLM, 10 mg of AML and 12.5 mg of HCT, separately in 50 mL acetonitrile. Stock solutions were stable for at least two weeks when stored refrigerated at 4°C.

### Preparation of pharmaceutical tablets sample solutions

Tribenzor® tablets were weighed and finely powdered. An accurately weighed portion of the powder equivalent to 40 mg of OLM, 13.9 mg of AML (equivalent to 10 mg of AML base) and 25 mg of HCT was extracted into acetonitrile with the aid of sonication and the extract was filtered. The filtrate was diluted with acetonitrile to obtain final concentrations of 200, 69.5 and 125 μg mL^−1^ for OLM, AML and HCT, respectively. 500 μL of Tribenzor® tablet solution were transferred into a 5 mL volumetric flask and diluted to the mark with acetonitrile to get a final concentration of OLM (20 μg mL^−1^), AML (6.95 μg mL^−1^) and HTZ (12.5 μg mL^−1^). Spectral acquisition and the calculations were performed in the same manner as described in **"Calibration procedures".**

### Calibration procedures

#### Spectrophotometric methods

Aliquots of standard solutions equivalent to 5–40 μg mL ^-1^ of AML and HCT, 2.5 - 40 μg mL ^-1^ of OLM were transferred separately into a series of 5-mL volumetric flasks and the solutions were diluted to the volume with acetonitrile and mixed well. The zero order absorption spectra of the prepared solutions were recorded from 200 to 400 nm against acetonitrile as a blank and stored in the computer. Absorbance was measured at 359 nm, 315 nm and 260.5 nm (isoabsorpitive point) for determination of AML, HCT and total concentration of HCT and OLM respectively (OLM can be determined by subtraction). The calibration graphs were constructed by relating the absorbance at mentioned wavelengths to the corresponding concentrations of AML, HCT and OLM (or HCT) respectively. The regression equations for the data were computed.

#### ANN method

**Figure 2:**
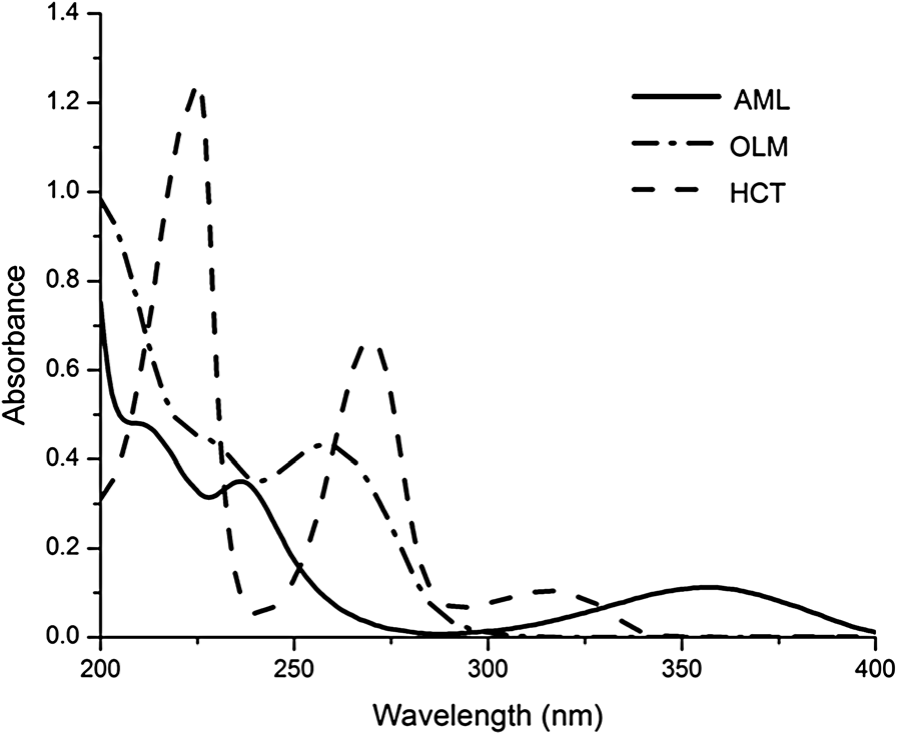
**Absorption spectra of AML, OLM and HCT against acetonitrile as a blank (10 μg mL**^**-1 **^**each).**

Five level, three factor calibration design [14] was used for construction of 25 samples by transferring different volumes of AML, OLM and HCT from their standard solutions into 5 mL volumetric flasks and the solutions were diluted to the volume with acetonitrile and mixed well (Table 1).16 samples were used to build the ANN model (training set) while 9 samples were used to test the predictive ability of the proposed models (validation set). The concentrations chosen for each compound in 25 samples were based on the calibration range of each of the two drugs, the ratio of AML: OLM: HCT in the Tribenzor tablets (1:4:2.5 respectively). The absorption spectra of the 25 samples were scanned from 200–400 nm against acetonitrile as a blank and transferred to Matlab for subsequent calculations. The noisy region from 200–230 nm and the zero absorbance of OLM and HCT after 340 nm accounted for the rejection of these parts from the spectra (Figure 2). Mean centering of the data proved to be the best preprocessing method for getting the optimum results.

**Table 1:** **The five level three factor experimental design of the training and validation set mixtures shown as concentrations of the mixture components in μg mL**^**-1**^

**Mix No.**	**OLM**	**AML**	**HCT**	**Mix No.**	**OLM**	**AML**	**HCT**
1	20	5	12.5	14	20	7	15
2	20	3	10	15	25	7	10
3	15	3	15	16	25	3	13.75
4	15	7	11.25	17	15	6	10
5	25	4	15	18	22.5	3	12.5
6	17.5	7	12.5	19	15	5	13.75
7	25	5	11.25	20	20	6	13.75
8	20	4	11.25	21	22.5	6	11.25
9	17.5	4	13.75	22	22.5	4	10
10	17.5	6	15	23	17.5	3	11.25
11	22.5	7	13.75	24	15	4	12.5
12	25	6	12.5	25	17.5	5	10
13	22.5	5	15				

### Assay of laboratory prepared mixtures by spectrophotometric methods

Aliquots of AML, OLM and HCT are transferred from their standard working solution into a series of 5-mL measuring flasks, completed to volume with acetonitrile to prepare mixtures containing different ratios of AML, OLM and HCT. The spectra of these mixtures are scanned from 200–400 nm and stored in the computer. For the determination of AML, the same procedures under *“Calibration procedures -spectrophotometric methods”* were applied. For the determination of HCT, the stored spectra of the laboratory prepared mixtures were divided by the spectrum of normalized AML (devisor), and the constant was subtracted from the ratio spectra followed by multiplication of the obtained spectra by the divisor. The result of these steps is the spectra of OLM and HCT binary mixture where HCT can be determined solely at 315 nm. Finally, absorbance was measured at 260 .5 nm (after removal of AML spectrum by ratio subtraction method) for determination of the total concentration of OLM and HCT (hence OLM by subtraction).

## Overview for ANN Method

Artificial neural network (ANN) is a type of artificial intelligence method that resembles biological nervous system in having the ability to find the relationship between inputs and outputs. ANN is composed of elements called artificial neurons that are interconnected by connections called weights. Commonly neural networks are trained, so that a particular input leads to a specific target output. The network is adjusted, based on a comparison of the output and the target, until the network output matches the target. Typically many input/target pairs are used to train a network [15].

ANN has a great advantage over other traditional multivariate methods in modeling linear and non-linear relationship between variables. There are many papers that describe the application of ANN on linear and non-linear data [16,17]. The type of ANN used in this paper is feed-forward model which was trained with the back propagation of errors learning algorithm. The back-propagation ANN is used in signal processing, data reduction and optimization, interpretation and prediction of spectra and calibration [17]. It is composed of three layers, an input layer in which the input data are introduced (e.g. absorbance in spectrophotometry). These inputs are passed to second hidden layer in which inputs are corrected and adjusted by weights and then finally passed to outer most layer (output layer) to give outputs (e.g. concentrations). The connections (weights) between layers are passed forward (from input to output layer), so it is called feed-forward ANN. The outputs (predicted concentrations) are compared with targets (actual concentrations) and the difference between them is called the error which is back propagated (and so called feed-forward ANN with the back propagation of errors learning algorithm) to network once more to be minimized through further adjustment of weights. ANN is iterated several times in such way till the error reaches a minimum value.

•Optimization of parameters of ANN

For proper training of ANN model, several parameters have to be optimized. There are two transfer functions used in ANN, one between input and output of a node in the hidden layer and the other is applied in output layer. The use of these functions depends on relationship between the inputs and outputs. Tan sigmoid followed by purelin are commonly used for non-linear systems while purelin-purelin transfer functions are used for linear one (as in our case). Among other ANN parameters, the hidden neurons number (HNN) is related to the converging performance of the output error function during the learning process. The learning coefficient (Lc) controls the degree at which connection weights are modified during the learning phase. The learning coefficient decrease (Lcd) and learning coefficient increase (Lci) control the variation of Lc value. It varies as a function of performance of the ANN (the Lc decreases or increases with the mean square error). For optimization of the ANN parameters, many experiments have to be done through which we can improve the model performance. Optimized ANN parameters are summarized in Table 2.

**Table 2:** Optimized parameters of ANN

** Parameter**	**Optimum value**
Architecture	110-10-3
Hidden neurons number	10
Transfer Functions	Purelin-purelin
Learning coefficient	0.001
Learning coefficient decrease	0.1
Learning coefficient increase	10

## Results and discussion

Tribenzor® tablets are combined dosage form containing the calcium channel blocker AML, the angiotensin II receptor blocker OLM, and the diuretic HCT. It has been used in the treatment of hypertension. The ratio of AML: OLM: HCT in tablets is 1:4:2.5 respectively. This study was designed to develop simple, robust and accurate spectrophotometric and ANN methods for the simultaneous determination of three analytes in Tribenzor® tablets. Because of the practical simplicity, and wide availability of spectrophotometry in quality control laboratories, it was attempted in this study.

### Spectrophotometric methods

**Figure 3:**
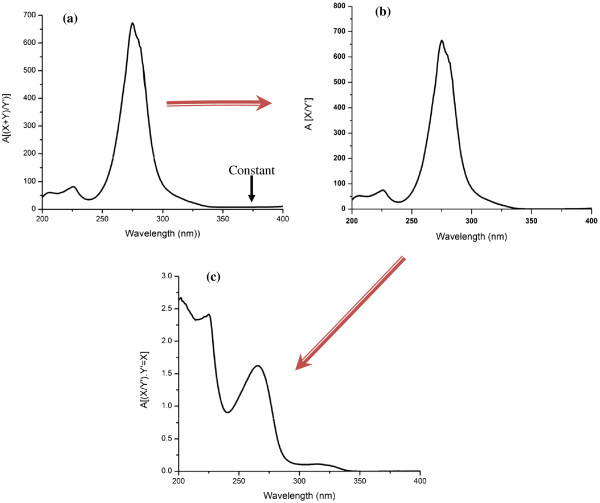
**(a) Ratio spectrum of laboratory prepared mixture of 7 μg mL**^**-1 **^**AML (Y) and 25 μg mL**^**-1 **^**OLM + 10 μg mL**^**-1 **^**HCT (X) using normalized AML (Y') as a divisor and acetonitrile as a blank (b) the same spectrum after subtraction of constant (c) the same spectrum after subtraction of constant then multiplication by the divisor (Y’).**

**Figure 4:**
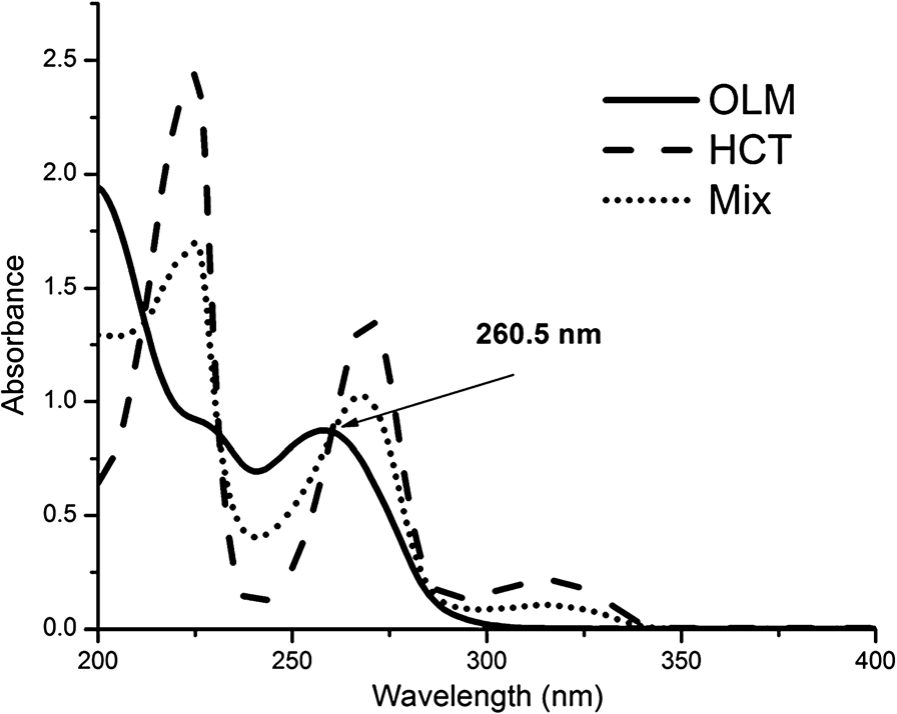
**Zero order absorption spectra of 20 μg mL**^**-1 **^**OLM, 20 μg mL**^**-1 **^**HCT and a mixture of 10 μg mL**^**-1 **^**OLM with 10 μg mL**^**-1 **^**HCT showing isoabsorptive point at 260.5 nm using acetonitrile as blank.**

The zero-order (D_0_) absorption spectrum of AML, OLM and HCT (10 μg mL^-1^ for each) solutions are recorded against acetonitrile as a blank over the range of 200–400 nm. The absorption spectra of the three compounds, AML, OLM and HCT show highly overlapped spectral band in the region 200–300 nm (Figure 2). AML can be determined solely at 359 nm directly where OLM and HCT show no absorbance. For determination of HCT and OLM, ratio subtraction method was applied [18] for removing AML spectrum. The method depends on that, when a mixture of OLM + HCT (X) and AML (Y) where the spectrum of (Y) is more extended (Figure 2), the determination of (X) could be done by scanning the zero order absorption spectra of the laboratory-prepared mixtures (AML and OLM + HCT), dividing them by carefully chosen concentration (normalized spectrum) of standard AML (Y' = divisor) producing a new ratio spectra that represent $\frac{X}{Y'}+\text{constant}$ (Figure 3a), then subtraction of the absorbance values of these constants (Y/Y') in plateau region (350–400 nm, Figure 3b), followed by multiplication of the obtained spectra by (Y') the divisor. Finally, the original spectrum of (X) could be obtained (Figure 3c) which is used for direct determination of HCT at 315 nm, where OLM shows no absorbance (Figure 2) and calculation of the concentration from the corresponding regression equation. The constant can be determined directly from the curve $\frac{X+Y}{Y'}$ by the straight line which is parallel to the wavelength axis in the region where (Y) is extended. For OLM assay, isoabsorptive method [19] was applied for determination of total concentration of OLM and HCT. Absorption spectra of 20 μg mL^-1^ OLM, 20 μg mL^-1^ of HCT, and of a mixture containing equal concentration of OLM and HCT (10 μg mL^-1^ of each) showed isoabsorptive point at 260.5 nm (Figure 4). By measuring the absorbance at the chosen isoabsorptive point in the zero order absorption spectrum obtained from ratio subtraction method, the total content of OLM and HCT in the mixture can be calculated, while the content of HCT alone can be calculated using the zero order absorption spectrum obtained from ratio subtraction method without any interference from OLM. Thus the content of OLM can be calculated by subtraction.

#### Methods validation

The proposed methods were validated according to the ICH-guidelines for validation of the analytical procedures [20] in terms of the linearity, sensitivity, accuracy, specificity, repeatability and reproducibility.

##### Linearity and sensitivity

A linear correlation was obtained between Absorbance and the corresponding concentrations of AML, HCT and OLM in the ranges of 5–40, 5–40 and 2.5-40 μg mL^−1^, respectively. The regression equations were:

For AML: A_359_ = 0.011C_1_ + 0.003(r = 0.9999)

For HCT: A_315_ = 0.010C_2_ + 0.001(r = 0.9999)

For OLM: A_260.5_ = 0.041C_3_ + 0.077(r = 0.9989)

where A was absorbance of the zero order absorption spectra, C_1_, C_2_ and C_3_ were the concentrations of AML, HCT and AML + OLM in μg mL^-1^, respectively, and r was the correlation coefficient. LOD and LOQ were calculated [20] according to the following equations:

$$\mathrm{LOD}=3\cdot \frac{3\sigma }{S\;\mathrm{and}\;\mathrm{LOQ}}=\frac{10\sigma }{S}$$

Where, σ was the standard deviation of the intercept of regression line and S was the slope of regression line of the calibration curve. The results are given in Table 3.

**Table 3:** Assay validation sheet of the proposed methods for the simultaneous determination of AML, OLM and HCT

***Parameter***	**Spectrophotometric methods**			**ANN method**		
	**Direct**	**Isoabsorptive**	**Ratio subtraction**			
	**AML**	**OLM**	**HCT**	**AML**	**OLM**	**HCT**
Accuracy (mean ± RSD)	99.89 ± 0.937	101.96 ± 0.622	99.55 ± 1.889	100.47 ± 1.197	99.9 ± 1.066	100.75 ± 2.072
Repeatability^a^	0.552	0.829	0.587	1.321	1.061	1.144
Intermediate precision^b^	0.925	1.327	1.593	1.119	1.683	2.424
Linear range (μg mL^-1^)	5-40	2.5-40	5-40	3-40	2.5-40	5-40
Slope	0.011	0.041	0.010	1.012	0.976	0.998
Intercept	0.003	0.077	0.01	−0.035	0.390	0.041
Correlation coefficient (r)	0.9999	0.9989	0.9999	0.9992	0.9995	0.9995
LOD (μg mL^-1^)	1.278	0.729	0.819	0.291	0.683	0.444
LOQ (μg mL^-1^)	4.217	2.209	2.482	0.880	2.070	1.345

^**a**^ The intraday (n = 3), average of concentrations; 6.95,20,12.5 μg mL^-1^for AML, OLM and HCT respectively, repeated three times within the day.

^**b**^ The interday (n = 3), average of concentrations; 6.95,20,12.5 μg mL^-1^for AML, OLM and HCT respectively, repeated three times in three days.

##### Accuracy

The accuracy of the proposed spectrophotometric methods was tested by analyzing triplicate samples of standard AML, HCT and OLM solutions. The recovery percentages were 99.89 ± 0.937, 99.55 ± 1.889 and 101.96 ± 0.622% for AML, HCT and OLM, respectively (Table 3). These results revealed the excellent accuracy of the proposed methods.

##### Repeatability and reproducibility

Intra-assay precision was assessed by analyzing 6.95 μg mL^−1^ of AML, 12.5 μg mL^−1^ of HCT and 20 μg mL^−1^ of OLM in triplicate in one assay batch. The inter-assay precision was assessed by analyzing the same concentrations in triplicate on 3 consecutive days. The average recovery percentages were around 100% and the low relative standard deviations (RSD) indicated the high accuracy and precision of the proposed methods, respectively (Table 3).

##### Specificity

AML, HCT and OLM were determined in laboratory prepared mixtures containing different ratios of the three drugs. The good recovery% and low standard deviations (SD) and relative standard deviations (RSD) proved the high specificity of the proposed methods (Table 4).

**Table 4:** Determination of AML, OLM and HCT in laboratory prepared mixtures by the proposed methods

**Concentration (μg mL**^**-1**^**)**			**Spectrophotometric methods**				**ANN method**	
			**Direct**	**Isoabsorptive**	**Ratio subtraction**			
			**Recovery % **^**a**^					
**AML**	**OLM**	**HCT**	**AML**	**OLM**	**HCT**	**AML**	**OLM**	**HCT**
7	25	10	101.56	101	101.81	101.62	98.2	102.56
5	15	13.75	98.55	100.36	103.8	101.05	100.01	101.2
6	15	10	97.27	98	103.54	100.07	97.28	100.01
7	15	11.25	98.96	100.2	103.03	102.44	101.84	102.58
7	17.5	12.5	101.56	102.26	100.89	99.55	98.11	100
5	17.5	10	102.18	100.52	102.22	100.75	100.35	100.89
6	17.5	15	100.30	100.66	103.73	101.93	101.89	100
5	20	12.5	100.36	97.71	102.06	101.34	100.99	100.35
6	20	13.75	100.91	98.82	101.34	101.22	99.9	100.47
Mean			100.18	99.24	101.89	101.11	99.84	100.90
SD			1.620	2.312	0.595	0.895	1.660	1.035
RSD%			1.617	2.33	0.584	0.885	1.663	1.026

^a^ average of three determinations.

### ANN method

ANN, as a multivariate calibration method, is very useful in spectral analysis because the simultaneous inclusion of many spectral wavelengths instead of using a single wavelength greatly improves the precision and predictive ability of the method [21]. The purpose of multivariate calibration method is to build a calibration model between the concentration of the analytes under study (AML, OLM and HCT in our case) and the experimental data (absorbance in our case). The first step in model building, involves constructing the calibration matrix for the tertiary mixture. In this study calibration set was optimized with the aid of the five level three factor design [14] resulting in 25 sample mixtures. Table 1 showed the composition of the 25 sample mixtures. Upon designing the calibration set, ratios of the three analytes in combined dosage has been taken into account. These 25 sample mixtures were splitted to 16 training mixtures (for building the models) and 9 validation mixtures (for measuring predictive power of the model). ANN model comprised of three layers: the input layer (absorbance matrix), the output layer (concentration matrix) and the hidden layer which consists of just single layer. One single hidden layer has been considered sufficient to solve similar or more complex problems. Moreover, more hidden layers may cause overfitting [16]. For proper modeling of ANN, different parameters should be optimized. These parameters are summarized in Table 2. From the most important parameters that should be optimized carefully, transfer function pair. Choosing of transfer function depends on the nature of data you work on. In our case, purelin-purelin transfer function was implemented in our models due to linear correlation between absorbance and concentrations. After optimization of parameters and architecture of the ANN, the training step is proceeded. ANN was trained by different training functions and there is no difference in performance (i.e. there is no decrease in root mean square error of prediction (RMSEP)). Levenberg–Marquardt training algorithm (TRAINLM) was thus preferred as it is time saving. To avoid overfitting, the validation set was involved in training step and ANN stops when RMSEP of calibration set decreased and that of independent set increased. To validate the predictive ability of the suggested model, ANN method was employed to predict the concentration of three analytes in nine laboratory-prepared mixtures (validation mixtures) containing different ratios of the three drugs where satisfactory results were obtained (Table 4). The predicted concentrations of the validation samples were plotted against the known concentrations to determine whether the model accounted for the concentration variation in the validation set. Plots were expected to fall on a straight line with a slope of 1 and zero intercept. AML, HCT and OLM, in all samples, lay on a straight line and the equations of these lines were y = 1.012 × − 0.035 (r = 0.9992) for AML, y = 0.976 × + 0.390 (r = 0.9995) for OLM and y = 0.998 × + 0.041 (r = 0.9995) for HCT. The three plots had a slope of almost 1 and an intercept close to zero. Other validation parameters for ANN method were presented in Table 3. The above mentioned methods were applied for analysis of AML, OLM and HCT in dosage form (Tribenzor® tablets). It was clear from Table 5 that all models were accurate and precise for AML, OLM and HCT determination. The results obtained by applying the proposed spectrophotometric and ANN methods for simultaneous determination of the three drugs in dosage form (Tribenzor® tablets) were statistically compared with those results obtained by the reference method [13]. It was concluded that with 95% confidence, there was no significant difference between the proposed and reference methods in terms of their accuracy and precision as the calculated *t −* and *F −* values were less than the theoretical values [22] (Table 5).

**Table 5:** Statistical comparison for the results obtained by the proposed methods and the reported method for the analysis of AML, OLM and HCT in Tribenzor® tablets (Batch No. 134809)

**Concentration (μg mL**^**-1**^**)**			**Spectrophotometric methods**			**ANN method**			**Reported method **^**a**^		
			**Ratio subtraction**	**Isoabsorptive**	**Direct**						
			**Recovery %**								
**AML**	**OLM**	**HCT**	**AML**	**OLM**	**HCT**	**AML**	**OLM**	**HCT**	**AML**	**OLM**	**HCT**
6.95	20	12.5	98.5	99.19	97.66	99.04	98.6	99.33	98.79	98.33	98.42
6.95	20	12.5	98.5	98.98	97.18	100.08	96.86	97	98.46	97.87	98.07
6.95	20	12.5	98.49	98.67	96.91	99.28	97.47	96.88	99.62	97.79	97.69
6.95	20	12.5	98.51	98.20	96.36	100.48	98.28	97.34	98.28	97.13	97.27
6.95	20	12.5	98.5	97.87	96.6	100.4	95.64	98.08	98.44	97.17	97.24
6.95	20	12.5	97.15	96.94	95.99	96.96	97.54	96.01	97.78	96.42	96.33
Mean			98.28	98.31	97.12	99.37	97.39	97.44	98.56	97.45	97.50
SD			0.552	0.829	0.587	1.321	1.061	1.144	0.615	0.678	0.736
RSD%			0.562	0.843	0.604	1.329	1.089	1.174	0.624	0.696	0.755
n			6	6	6	6	6	6	6	6	6
Variance			0.305	0.687	0.345	1.745	1.126	1.309	0.378	0.46	0.542
Student's t test^b^			0.842 (2.228)	1.955 (2.228)	0.994 (2.228)	1.608 (2.228)	1.355 (2.228)	1.73 (2.228)	-----	-----	-----
F value ^b^			1.241 (5.050)	1.497 (5.050)	1.574 (5.050)	4.307 (5.050)	3.338 (5.050)	1.574 (5.050)	-----	-----	-----

^**a**^ Reference spectrophotometric method is that published in the literature [13].

^**b**^ The values in the parenthesis are the corresponding theoretical values of t and F at P = 0.05.

## Conclusion

From the previous discussion, it could be concluded that the proposed procedures are simple, rapid over chromatographic methods and it does not need sample preparation or sophisticated techniques and instruments. From the economic point of view, the analytical reagents are inexpensive and available in all laboratories. The validation of the proposed methods according to the ICH guidelines proved the applicability and great value of these methods for routine application in quality control laboratories for the simultaneous analysis of AML, OLM and HCT in their pure powder and dosage forms without prior separation or excipient interference.

## Competing interests

The author declares that he has no competing interest.
